# A rare case of Behçet’s disease complicated by Budd-Chiari syndrome and perforated duodenal ulcers in a young male

**DOI:** 10.1093/omcr/omae135

**Published:** 2024-11-20

**Authors:** Osama Hroub, Kareem Ibraheem, Abdalrahman N Herbawi, Mohammad Hroub, Mohammad I Smerat, Ahmad Batran

**Affiliations:** Faculty of Medicine, Palestine Polytechnic University, Hebron 9020000, Palestine; Faculty of Medicine, Palestine Polytechnic University, Hebron 9020000, Palestine; Faculty of Medicine, Palestine Polytechnic University, Hebron 9020000, Palestine; Faculty of Medicine, Palestine Polytechnic University, Hebron 9020000, Palestine; Radiology Department, Al-Ahli Hospital, Hebron 902000, Palestine; Faculty of Medicine, Palestine Polytechnic University, Hebron 9020000, Palestine

**Keywords:** Behçet’s disease, vasculitis, Budd-Chiari syndrome, kissing ulcer, duodenal ulcer, liver transplant

## Abstract

Behçet’s disease (BD) is characterized by skin lesions, uveitis, and recurrent oral and genital ulcers. Vascular problems, predominantly affecting veins, lead to thrombosis, increasing the risk of ruptured artery aneurysms and Budd-Chiari syndrome (BCS). Morbidity and mortality are significantly heightened by rare occurrences such as pulmonary artery aneurysms, cardiac involvement, and BCS. Prompt diagnosis and treatment are pivotal for prognosis improvement, particularly in males with early onset. We present a case of a 16-year-old male with BD history, who developed abdominal distension, pedal edema, and shortness of breath. Clinical examination and laboratory findings revealed thrombosis in the right popliteal vein and BCS. Despite the initiation of lifelong anticoagulation therapy, the patient later suffered a gastrointestinal bleed from perforated duodenal ulcers, necessitating emergency intervention. Given a high Model for End-Stage Liver Disease (MELD) score and associated mortality risk, the patient was promptly referred for liver transplantation.

## Introduction

Behçet’s disease (BD) is a systemic vasculitis of uncertain origin, featuring a chronic and recurrent course. It is characterized by genital ulcers, oral aphthous ulcers, uveitis, and occasional vasculitis [1]. Reports indicate that as much as 17% of mortality in BD is connected to venous thrombosis, specifically involving conditions like pulmonary embolism or Budd-Chiari syndrome [2]. The term ‘Budd-Chiari Syndrome’ (BCS) loosely refers to obstruction in the outflow of hepatic veins [3]. Budd-Chiari syndrome (BCS) is a rare and severe complication of BD involving thrombosis in the hepatic veins and/or the intrahepatic or suprahepatic inferior vena cava. The significant vascular complications linked with BCS in BD appear to predominantly impact young males [4]. Herein, we present a 16-year-old male with a history of BD complicated by BCS who presented with severe epigastric pain, tarry black stools, nausea, and vomiting. He was diagnosed with two kissing ulcers in the second part of the duodenum by endoscopy.

## Case presentation

**Figure 1:**
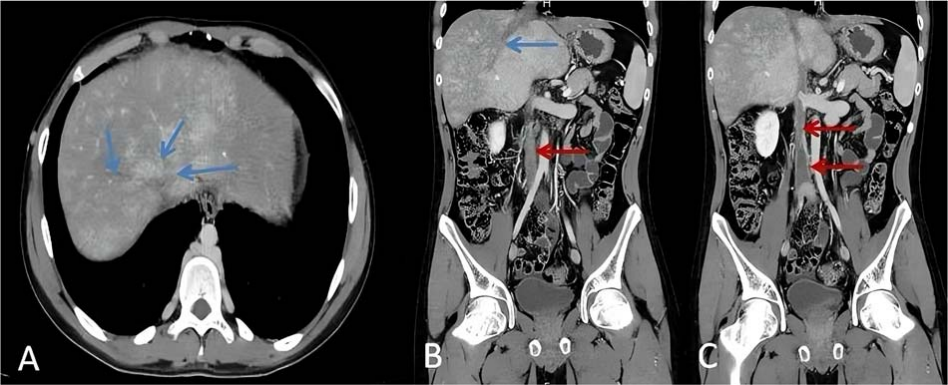
Axial and coronal CT image in a patient with BCS shows non-opacified hepatic veins (blue arrows, A and B). Coronal CT image in a patient with thrombosis of IVC seen as filling defect causing expansion of the lumen (red arrows, B and C).

A 16-year-old male presented with a 4-month history of abdominal distension, pedal edema, and shortness of breath. He reported experiencing right leg pain and a rash in the days preceding his current presentation. He was diagnosed with Behçet’s disease (BD), characterized by oral and genital ulcers, two years prior, based on international criteria, and had been stable on azathioprine and prednisone without flares until 4 months ago. There were no known risk factors predisposing him to thrombosis. Physical examination revealed right popliteal tenderness and warmth, as well as tenderness in the right upper quadrant, hepatomegaly, and ascites. Laboratory findings showed hemoglobin of 13.1 g/dL, MCV 79 mg/dL, platelets 23 040/mm^3^, WBCs 7000/μL, RBS 102 mg/dL, elevated liver enzymes (ALT 78 U/L, AST 47 U/L, ALP 134 U/L, GGT 83 U/L, bilirubin 0.9 mg/dL, direct bilirubin 0.5 mg/dL), INR 1.6, PT 15 seconds, elevated acute phase reactants (ESR 35 mm/h, CRP 32 mg/L), and a D-Dimer of 1460 ng/ml. Doppler ultrasonography revealed thrombosis in the right popliteal vein. An abdominal CT scan with IV contrast revealed a filling defect in the inferior vena cava (IVC), indicating thrombosis extending from its intrahepatic segment down to its bifurcation into the common iliac veins and the hepatic veins. This venous occlusion is attributed to thrombosis in the hepatic veins and the IVC (Fig. 1), suggesting Budd-Chiari syndrome.

He began lifelong low-molecular-weight heparin therapy. Additionally, he underwent a pulmonary CT angiogram to exclude any vascular complications, with no abnormalities detected.

The patient then underwent esophagogastroduodenoscopy as part of gastrointestinal follow-up, yielding normal results. However, he returned to the hospital two months later with severe epigastric pain, nausea, vomiting, and black stools for 24 h. Upon assessment, he appeared pale, jaundiced, and dehydrated, with a tender abdomen. Laboratory results indicated a drop in hemoglobin to 7 g/dL, along with elevated creatinine, bilirubin, and INR. During an emergency endoscopy, bilateral kissing ulcers were found in the second part of the duodenal (D2), and bleeding was controlled with epinephrine injections and clips. The patient received blood transfusions, intravenous fluids, and intravenous proton pump inhibitors (PPIs). His vitals improved, and his hemoglobin level rose to 11.4 g/dL after hemostasis during the endoscopic procedure and blood transfusion. Considering the patient’s MELD score of 23 and an estimated 19.6% mortality risk within three months, the decision was made to promptly refer him for liver transplantation.

## Discussion

Behçet’s disease (BD), a multisystem inflammatory condition of uncertain cause, manifests with recurrent genital and oral ulcers, uveitis, and skin lesions [4]. It often follows a relapsing course, with vascular involvement occurring in 5%–40% of cases, mainly affecting veins due to vascular inflammation rather than thrombophilic reasons [5]. Thrombosis in BD may be associated with deficiencies in protein C, protein S, and antithrombin III, as well as the presence of antiphospholipid antibodies, factor V Leiden, and the prothrombin 20210A mutations [4].

Patients with vasculo-Behcet are prone to progressive multifocal vessel-related problems and recurrent vascular lesions, with the aorta having the highest risk of developing aneurysms, followed by the pulmonary, femoral, subclavian, cervical, and popliteal vessels. The acute arterial complications in particular need to be managed as an emergency [1].

Male patients are at greater risk than female ones of developing vascular involvement. As in our case, men and patients with a younger age of onset are associated with a more severe outcome [6].

Uncommon vascular consequences such as Budd-Chiari syndrome (BCS), cardiac involvement, and pulmonary artery aneurysms are associated with high morbidity and mortality. BCS presents in two forms: symptomatic, with a mortality rate up to 60%, characterized by abdominal pain, ascites, and abdominal wall collateral circulation; and silent, with a 10% mortality rate, marked by the absence of ascites and effective collateral formation [5].

Inferior vena cava (IVC) blockage, which causes BCS, represents a rare manifestation of vein involvement, whereas major artery involvement is more prevalent. Both artery aneurysm rupture and BCS contribute to a high death rate [1]. BCS arises from obstruction of intrahepatic or suprahepatic veins. Evidence from regions where Behçet’s syndrome (BS) is prevalent suggests that BCS should be considered as a potential diagnosis in cases of IVC obstruction [4].

Medical interventions such as anticoagulation, vasculitis therapy, and diuretics, as needed, can improve the prognosis. Some experts who advocate for exclusive management with immunosuppressive agents recommend monthly cyclophosphamide pulses combined with prednisolone at a dosage of 1 mg/kg, followed by tapering of prednisolone to fewer than 10 mg/day after three months [4]. In cases that are resistant, anti-tumor necrosis factor (TNF) agents may also be effective [5]. In the subset of patients with BCS who undergo step-by-step treatment, about 10% to 20% experience failure of anticoagulation, angioplasty, or transjugular intrahepatic portosystemic shunt (TIPS) due to either technical issues or unsatisfactory clinical outcomes despite technically successful procedures, necessitating rescue transplantation [7].

Regarding the gastrointestinal involvement in BD, patients either had isolated gastric, isolated duodenal, or combined gastroduodenal ulcers [8]. Ulcerations can occur throughout the intestine, with the ileocecal region being the most common site. However, the true prevalence of ileocecal ulcers requires further observation [8, 9]. In our case, emergency endoscopic intervention with clips and blood transfusion was required for the management of perforated duodenal ulcers.
